# Towards Blue Long-Lasting Luminescence of Eu/Nd-Doped Calcium-Aluminate Nanostructured Platelets via the Molten Salt Route

**DOI:** 10.3390/nano9101473

**Published:** 2019-10-16

**Authors:** Rocío E. Rojas-Hernandez, Fernando Rubio-Marcos, Aida Serrano, Eduardo Salas, Irina Hussainova, José Francisco Fernandez

**Affiliations:** 1 Electroceramic Department, Instituto de Cerámica y Vidrio, CSIC, Kelsen 5, 28049 Madrid, Spain; frmarcos@icv.csic.es (F.R.-M.); aida.serrano@icv.csic.es (A.S.); jfernandez@icv.csic.es (J.F.F.); 2 Department of Materials Engineering, Tallinn University of Technology, Ehitajate 5, 19180 Tallinn, Estonia; irina.hussainova@taltech.ee; 3 Escuela Politécnica Superior, Universidad Antonio de Nebrija, C/Pirineos, 55, 28040 Madrid, Spain; 4 Spanish CRG BM25 SpLine beamline, The European Synchrotron, 38043 Grenoble, France; edusc22@gmail.com; 5 Instituto de Ciencia de Materiales de Madrid, CSIC, 28049 Madrid, Spain

**Keywords:** calcium-aluminates, molten salt method, persistent luminescence, XANES, phosphor, rare-earth

## Abstract

Calcia-alumina binary compounds doped with rare earths and some transition metals cations show persistent luminescence from the visible to the infrared range. Specifically, the blue light can be obtained through the Eu^2+^ activator center in a potential host, such as dodecacalcium hepta-aluminate (Ca_12_Al_14_O_33_) and monocalcium aluminate (CaAl_2_O_4_). By doping with Nd^3+^, the persistent luminescence can be substantially prolonged; for this reason, the Eu/Nd pair is a potential choice for developing long-lasting blue luminescence. Herein, the phase evolution of the calcia-alumina system via molten salt synthesis is reported as a function of the synthesis temperature and the atmospheric environment. The fraction of CaAl_2_O_4_ phase increases when the temperature is higher. Synthesized microparticles of platelet-type morphology represent isolated nanostructured ceramic pieces. Under visible light, the particles are white. This indicates that the followed process solves the dark-gray coloring of phosphor when is synthesized in a reduced atmosphere at high temperature. As regards the synthesis mechanism, which is assisted by the molten flux, the dissolution−diffusion transport process is promoted at the surface of the alumina microparticles. In fact, the emission intensity can be modulated through the phase of the Eu-doped calcium-aluminate discrete platelets synthesized. Consequently, the photoluminescence intensity depends also on the oxidation state of the Eu ion. X-ray absorption near-edge structure and photoluminescence measurements corroborate the Eu reduction and the grain coarsening with the enhancement of the blue emission. The doped phosphors with Eu/Nd show a broad and strong absorption in the region of 320–400 nm and a broad emission band at around 440 nm when they are excited in this absorption range. From a broader perspective, our findings prove that the Ca_12_Al_14_O_33_ and CaAl_2_O_4_ phases open new opportunities for research into the design of blue long-lasting emitters for a wide range of fields from ceramic to optoelectronic materials.

## 1. Introduction

Research on rare earth luminescent materials has been stimulated over the last decade by several important industrial applications, such as bioimaging and energy harvesting. There is a great demand for rare earth-doped oxide materials with excellent luminous efficiency and high thermal stability. Specifically, alkaline-earth metal aluminates are oxides that fulfill these requirements [1], as such materials have the unique advantages of high quenching temperature, corrosion resistance, low cost and non-toxicity, which makes them economically attractive. Nowadays, not only the emission of light in a fixed range (Ultraviolet, (UV), to Near Infrared Region, (NIR)) is required, but also the capability of materials to act as an optical storage is needed. These materials should emit after the removal of the activation light source. A wide range of applications, including but not limited to, devices for a night vision, optical storage, solar energy, and in-vivo imaging, is opened for such kinds of these long-lasting luminescent materials. The well-known persistent compound SrAl_2_O_4_:Eu,Dy can emit in the green color for up to 24 h [2,3]. Although the research is focused mainly upon green emission due to the large sensitivity in this region of the human eye (luminous efficacy) in light and dark conditions, the persistent emission other wavelength ranges (blue or red light emission) is also desirable. 

There are specific applications that demand persistent blue and red phosphors with comparable emission durations to those of green ones. It is worth remarking that UV-chip or UV light can activate the phosphors, but the sunlight activation can represent a great achievement due to the reduction of energy consumption. Therefore, evaluation of the efficiency of the persistent phosphors activated by natural sunlight is very much in demand. 

The persistent blue-emitters materials have usually been based upon the strontia-alumina (SrO-Al_2_O_3_) system, such as Sr_2_Al_6_O_11_, Sr_4_Al_14_O_25_, SrAl_4_O_7_, etc. [1]. The best candidate for the blue long persistent emitter is the outstanding CaAl_2_O_4_: Eu^2+^ [1], where co-doping it with Nd^3+^ enhances the afterglow duration due to the depth increment [4]. Although the photoluminescence (PL) mechanism has not yet been established by consensus, the studies indicate that the oxygen vacancies are the main trap centers for the mono-doped (Eu) host, and the co-doping with Nd^3+^ increases the electron carriers and feeds deeper traps [5,6]. CaAl_2_O_4_ and Ca_12_Al_14_O_33_ phases are the most studied ones; they have been synthesized by different methods including combustion, sol-gel, co-precipitation and solid-state approaches [6,7,8,9]. However, the produced materials usually contain undesirable phases such as CaAl_4_O_7_ and Ca_3_Al_2_O_6_. The reaction processes are not trivial in the CaO-Al_2_O_3_ system, and many intermediated phases can be generated [8]. The processing of these materials requires two or three steps at a high temperature, a quite long reaction time and annealing in a reductive atmosphere. Moreover, the particles obtained by these routes tend to agglomerate, forming small clusters with non-uniform shapes and sizes [8,10], which is not suitable for practical applications. 

Our previous study related to the synthesis of the long-lasting green luminescent materials based on SrAl_2_O_4_: Eu, Dy has demonstrated that the starting alumina plays a key role in the kinetics of the reaction when a molten flux is employed. This reactivity is modulated by the nature, size, and morphology of the precursor. It is possible to synthesize the green emitters, SrAl_2_O_4_: Eu, Dy, with a pseudo-spherical morphology and a particle size ≤ 0.5 µm when a sub-micron Al_2_O_3_ (0.1 µm Al_2_O_3_) is employed [11,12]. However, the growth of SrAl_2_O_4_: Eu, Dy sub-micron particles on the surface of the hexagonal platelets of 6µm Al_2_O_3_ is promoted when a larger alumina particle, 6 µm Al_2_O_3_, is employed, modifying the reaction pathway and leading to a different reaction evolution. One of the advantages of the platelets obtained is their thickness of ca. ≤ 1 µm, which is suitable for potential applications. Here, as the alumina precursor, 6 µm Al_2_O_3_ particles were employed to study the reaction pathway to synthesize calcium aluminate luminescent compounds, obtaining persistent emission in the blue range. A key parameter in the molten salt method is the nature of the molten salt flux. There are several requirements for the nature of the salt that should be fulfilled, among them, there is an adequate melting point (m.p.), good chemical stability, ease of washability, and cots effectiveness. 

The phase formation of the desired CaAl_2_O_4_ is expected to occur at high temperatures; for this reason the eutectic mixture of NaCl-KCl (the melting point of which is 659 °C) was selected, also taking into consideration the high solubility in glycerol of chlorides [13], which may be important for easy removal of the NaCl-KCl mixture if the salts remain during the synthesis treatment, avoiding the water exposure.

Herein, we present a practically scalable approach for the synthesis of blue emitters based on Ca_12_Al_14_O_33_ and CaAl_2_O_4_. Through this novel method, we have found a linked relationship between the phase composition and the photoluminescence intensity. These results open prospects for a molten salt route to be considered as a promising technique for the preparation of complex oxide ceramics such as aluminates.

## 2. Materials and Methods 

### 2.1. Synthesis

The calcium aluminates (CAO) particles were produced by a molten salt-assisted route. The starting materials were aluminum oxide (aka alumina, Al_2_O_3_) (Almatis, Paris, France, Specific Surface Area, BET: 13 m^2^/g, average particle size, d_50~_6 μm), calcium carbonate (CaCO_3_) (Merck, Burlington, MA, USA, 99.9%, d_50_~1.1 μm,), europium(III) oxide (Eu_2_O_3_), (Metal Rare Earth Limited, Shenzhen, China, 99.5%, d_50_~3.8 μm) and neodymium(III) oxide or neodymium sesquioxide (Nd_2_O_3_) (Rare Earth Limited Shenzhen, China, 99.5%, d_50_~3.1 μm). The stoichiometric mixture of raw materials to obtain the Ca_1-*x*-*y*_Eu*_x_*Nd*_y_*Al_2_O_4_ composition with *x* = 0.02 and *y* = 0.01 is used. The precursors were dried at 120 °C for 1 h before dry homogenization by grinding in a 60 cm^3^ nylon container for 20 min by using a turbula-type mixer at 50 rpm with zirconium dioxide (aka zirconia, ZrO_2_), balls of 0.5 mm diameter. The mixed CAO and CAO: Eu^2+^, Nd^3+^ powders were then annealed at temperatures in the range of 1000 to 1400 °C in a tube furnace in an air atmosphere and under a 90N_2_-10H_2_ reducing atmosphere, respectively, and finally sieved by using a 100 μm sieve. 

### 2.2. Structural and Microstructural Characterization

The crystalline phases were characterized by X-ray diffraction (XRD, D8, Bruker Corporation, Billerica, MA, USA) using a Lynx Eye detector and Cu Kα_1,2_ radiation. The crystallite size (*D*) of the powders can be estimated from the full width at half maximum of the diffraction peak by the Scherrer Equation:

(1) $$D=\frac{K\lambda }{B\cos\theta }$$

where *λ* is the X-ray wavelength, B the half maximum of the diffraction peak, θ is the angle of diffraction, and using a shape factor (*K*) of 0.9.

Particle size distribution measurements of the phosphor powders were performed by laser diffraction (Mastersizer S, Malvern, Worcestershire, UK). The morphology of powders was evaluated using secondary electron images of a field emission scanning electron microscopy (FE-SEM, Hitachi S-4700, Marunouchi, Chiyoda-ku, Tokyo, Japan). The average grain size of the calcium aluminate phases formed during the thermal treatment was calculated from FE-SEM micrographs by an image processing and analysis program (Leica Qwin, Leica Microsystems Ltd., Cambridge, England) considering more than 50 grains in each measurement.

Room temperature X-ray absorption near-edge structure spectroscopy (XANES) measurements of powders at the Eu L_3_ edge were performed in fluorescence mode detection at 45° incidence at the Spanish CRG beamline BM25A (SpLine) at The European Synchrotron (The ESRF), in Grenoble (France). The signal was measured using a 13 element Si (Li) solid-state detector from e2V Scientific Instruments ( Sirius House, Watery Lane, Wooburn Green, High Wycombe, Buckinghamshire, UK). The conclusive spectra represent an average of three X-ray absorption spectroscopy (XAS) scans. The XAS data were analyzed using ATHENA software (Demeter 0.9.26). [14] 

### 2.3. Luminescent Characterization

The excitation and emission spectra of the synthesized CAO particles were recorded using a spectrofluorometer (Fluorolog^®^-3, HORIBA Jobin Yvon, Neuhofstraße 9, 64625 Bensheim, Germany) equipped with a 450 W xenon arc lamp as the light source at room temperature. The afterglow decay curves were measured after irradiating the CAO particles with Ultraviolet, (UV) light at 365 nm for 10 min. 

## 3. Results & Discussion

This section may be divided by subheadings. It should provide a concise and precise description of the experimental results, their interpretation as well as the experimental conclusions that can be drawn.

### 3.1. Understanding the Phase Composition in CaO-Al_2_O_3_ System as a Function of the Temperature and Atmosphere Conditions

As a first approximation, we evaluated the vast synthesis conditions to facilitate the formation of the desired crystalline structure. For this, the CAO phosphors were synthesized employing the molten salt method by being heated from 1000 to 1400 °C for 2 h in an air atmosphere, employing a salt/CAO molar ratio of 3:1 and an Al_2_O_3_/CaO ratio of 1, using alumina of an average particle size d_50~_6 μm. Regarding the amount of salt to be used, it is usually comprised between 80 and 120% in the weight of the reactant mixture, or using molar ratio salt/complex oxide (S/O), S/O = 1:1, 3:1, 4:1, 5:1 or 20:1 [15]. After an experiment series, we found that the ratio 3:1 offers the most suitable synthesis conditions, analogously to synthesis for the SrAl_2_O_4_ phosphor. 

**Figure 1:**
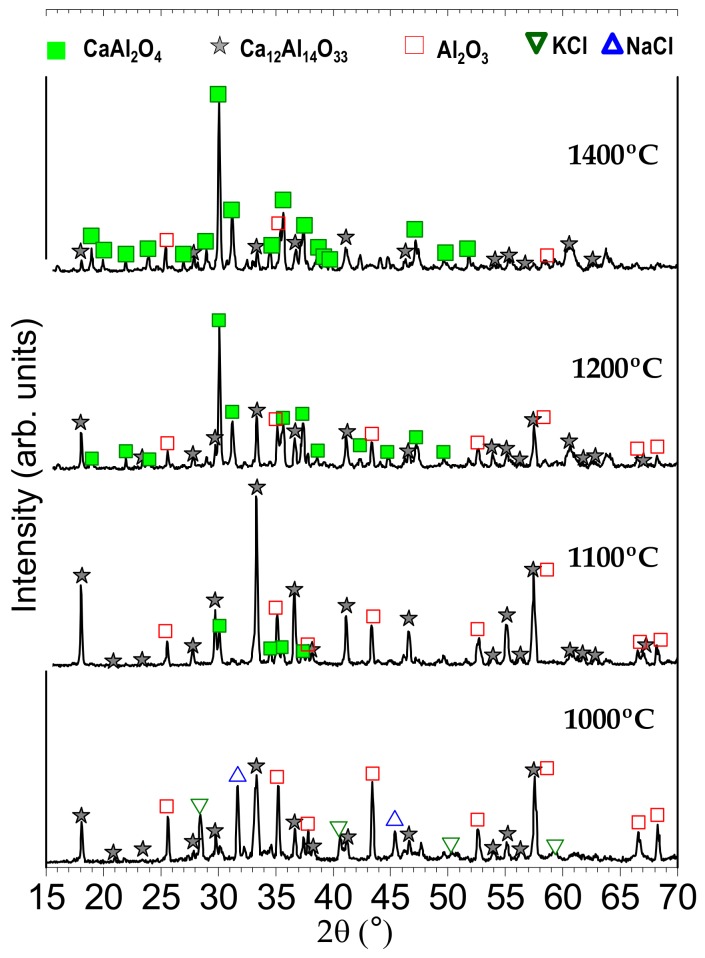
X-ray diffractograms (XRD) of synthesized calcium oxide (CaO): Eu, Nd phosphor heated at 1000, 1100, 1200 and 1400 °C for 2 h in air atmosphere, employing a salt/CAO molar ratio of 3:1. The symbols highlight aluminum oxide (Al_2_O_3_) (red-open squares), sodium chloride (NaCl) (blue-open triangles), potassium chloride (KCl) (green-open triangles), monocalcium aluminate (CaAl_2_O_4_) (green squares) and dodecacalcium hepta-aluminate (Ca_12_Al_14_O_33_) (gray stars).

Figure 1 shows the XRD pattern of the powder synthesized at 1000, 1100, 1200 and 1400 °C in the air atmosphere. At 1000 °C, the XRD shows characteristic peaks of the Ca_12_Al_14_O_33_ cubic polymorph, whose pattern is characterized by four peaks centered in the 2θ axis at 18.11°, 27.82°, 33.39° and 41.20°, ascribed to the (211), (321), (420), (521) facet diffraction of the cubic, and matched with the Ca_12_Al_14_O_33_ standard values given in JCPDS (No. 09-0413 or 70-2144) and their coexistence with the NaCl (JCPDF file 72-1668) and KCl (JCPDF file 76-3376) phases. From the CaO-Al_2_O_3_ diagram [16], the Ca_12_Al_14_O_33_ phase has the lowest forming temperature, around 1400 °C. Applying the molten salt synthesis, this forming temperature can be reduced down to be around 1000 °C, as is shown in Figure 1. In addition, the XRD reveals the presence of another phase, which could be identified as Al_2_O_3_ (JCPDF file 73-1512). The intensity of the XRD peaks of the salt decreases with an increase in temperature, due to its vaporization during the thermal treatment. As commented above, the melting point of the eutectic mixture is observed at 659 °C; however, the remaining salts could be related to the presence of pure salts into the melt. In theory, the 100% weight loss of the eutectic mixture occurs at ca. 1010 °C, but a shift to a higher temperature is expected when other compounds are added into the system, as it has already reported in a previous work [12]. 

At 1100 °C, the main phase Ca_12_Al_14_O_33_ coexists with the monoclinic CaAl_2_O_4_ phase (JCPDS 70-0134) characterized by the diffraction peaks at 2θ = 18.99°, 30.14°, 35.42°, and 37.179°, attributed to the (−112), (220), (006), (313) crystal facets. At 1200 °C, the amount of monoclinic CaAl_2_O_4_ phase increases, reaching the maximum of CaAl_2_O_4_ fraction at 1400 °C. Taking into account the reaction mechanism established previously [12], the most probable origin of the rest of these raw materials can be related to an unreacted Al_2_O_3_ core. From these observations, it can be stated that a thermal treatment at 1000 °C produces the Ca_12_Al_14_O_33_ compound, and the CaAl_2_O_4_ compound is synthesized at 1400 °C. Between 1000 and 1400 °C, there is a coexistence of both phases. In the case of calcium aluminate host materials, it is difficult to produce the pure phase products owing to the generation of many phases together during the preparation, so the presence of the mixed phases may influence the luminescence performance of rare earth compounds. 

**Figure 2:**
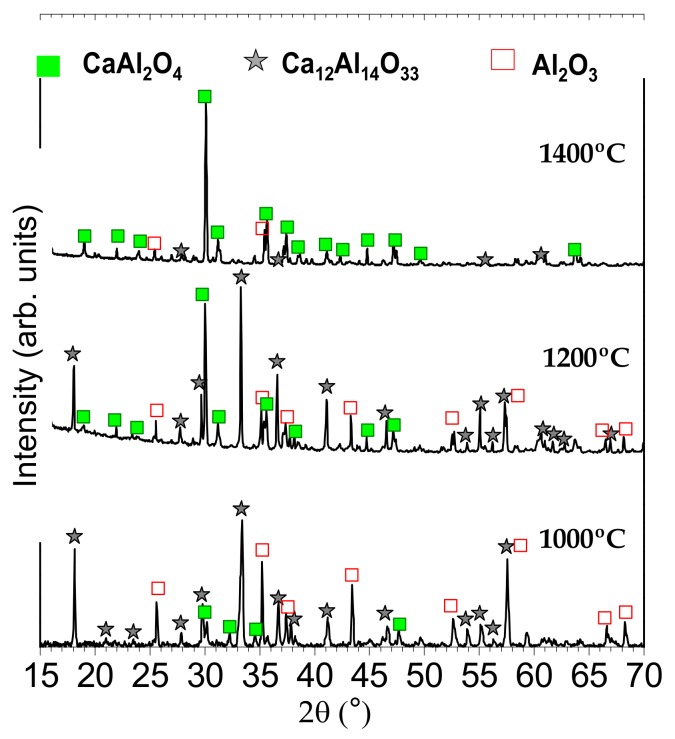
X-ray diffractograms of synthesized CAO: Eu, Nd phosphors heated at 1000, 1200 and 1400 °C for 2 h in 90N_2_-10H_2_, employing a salt/CAO molar ratio of 3:1. The symbols highlight Al_2_O_3_ (red-open squares), CaAl_2_O_4_ (green squares) and Ca_12_Al_14_O_33_ (gray starts).

In order to obtain a phosphorescence response, europium and neodymium were incorporated as the dopants to synthesize CAO: Eu^2+^, Nd^3+^ materials. The powders were thermally treated at 1000, 1200 and 1400 °C for 2 h in a furnace under a nitrogen–hydrogen (90N_2_–10H_2_) atmosphere to reduce Eu^3+^ to Eu^2+^. As shown in Figure 2, the XRD pattern of the powder, which was thermally treated at 1000 °C for 2 h, is similar to that annealed in air. In both cases the main phase is Ca_12_Al_14_O_33_. By contrast, under a reducing atmosphere, there is no residual salt revealed. Taking into account in the thermogravimetric (TG) analyses presented in detail elsewhere [12], the salt starts to vaporize above the m.p. (659 °C), and it is expected that the 100% weight loss of the eutectic mixture occurs at ca. 1010 °C. However, the other components in the system can delay the total evaporation of the salt. During the thermal treatment of samples, a continuous gas flow of 90N_2_-10H_2_ at the pressure of 1 bar is used in a tubular furnace. 

Thus, it may be expected that the gas flow passing through the chamber promotes the dragging of the salt during the processing time, removing the remains of salt when CaO and Al_2_O_3_ are incorporated. At 1200 °C, there is a coexistence of Ca_12_Al_14_O_33_ and CaAl_2_O_4_ phases with the minor amount of alumina Al_2_O_3_. Increasing the temperature up to 1400 °C results in the formation of the CaAl_2_O_4_ phase as a principal phase accompanying a trace amount of the Al_2_O_3_ phase. The crystallite size was also calculated; being 44(2), 55(3) and 63(3) nm for the samples synthesized at 1000, 1200 and 1400 °C, respectively. As expected, the crystallite size increases as the sintering temperature increases, indicating that the crystallinity of the CAO phase is increased.

Therefore, the development of Ca_12_Al_14_O_33_ and CaAl_2_O_4_ phases by the molten salt route at 1000 and 1400 °C, respectively, can be stated. Both polymorphs are the potential candidates to emit in the blue range conditioned by the successful incorporation of the doping elements.

### 3.2. Effect of the Heat Treatment on the Morphology, and Particle Size Distribution

**Figure 3:**
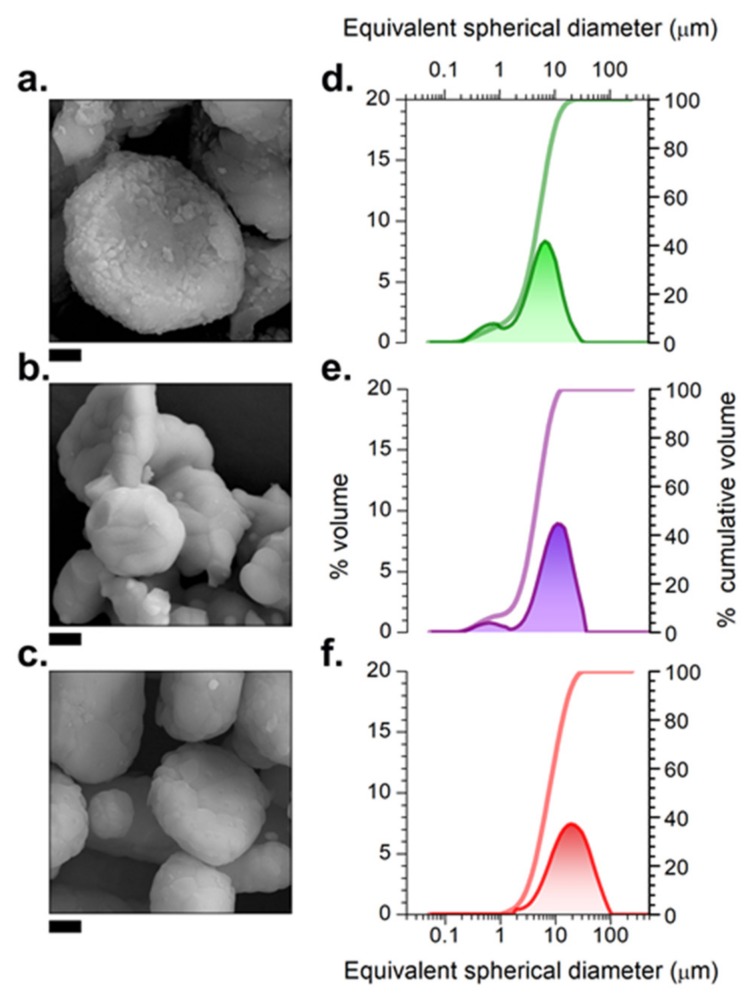
Field-emission scanning electron microscopy (FE-SEM) micrographs of the synthesized particles based on CAO: Eu, Nd phosphor heated at (**a**) 1000, (**b**) 1200 and (**c**) 1400 °C for 2 h in 90N_2_-10H_2_, employing a salt/CAO molar ratio of 3:1. Scale bars in panels (**a**) and (**b**) correspond to 1 μm, while in panels (**c**) they correspond to 2 μm. The particle size distribution of the particles synthesized at (**d**) 1000, (**e**) 1200 and (**f**) 1400 °C.

Figure 3a–c depict the FE-SEM micrographs of the synthesized CAO particles, obtained from 1000 to 1400 °C. The morphology of the platelet-like shaped alumina micro-particles is preserved at all temperatures of treatment. The alumina acts as a template, promoting the dissolution−diffusion transport mechanism of calcia and the rare-earth dopants by the eutectic nature of the salt mixture [12]. The procedure described allows synthesizing particles to possess a particle size in the micrometric range. Specifically, the particle size refers to the platelet-like shaped CAO microparticles. Due to the powder being not monodisperse, the particle size distribution describes the poly-dispersity character, reflecting the agglomeration state of these microparticles (see Figure 3d–f, and Table 1). The microparticles are also nanostructured, and the synthesis of CAO: Eu, Nd occurs at the surface. The eutectic flux dissolves the CaO and transports this reactant to the surface of Al_2_O_3_. These platelets-like particles have a particle size smaller than 10 µm for the samples synthesized at 1000 and 1200 °C and ca. 17 µm for the sample obtained at 1400 °C that exhibits an additional formation of sintering necks between platelet particles through the CAO phase. 

These nanostructured platelet-like particles are composed of grains or primary particles, which evolve as a function of the synthesis temperature as a consequence of the coalescence and grain growth process, as is shown in Figure 3. At 1000 °C the average grain size is ranged 100–500 nm, and a second phase is located between the grains. 

At 1200 °C the average grain size increases up to 800–1200 nm, and in this case, the shape of the grains approaches straight grain boundaries and triple point junctions with 120° indicating a near-equilibrium microstructure. However, the heat treatment at 1400 °C promotes the grain growth in each individual microparticle to get grains ranging from 1 up to 2 µm. The preferential growth occurs in the platelet plane because the thickness of the CAO platelets does not exceed 2 µm, which is a beneficial dimension for applications. As the main phase at a higher temperature is CaAl_2_O_4_, it is possible to correlate the grain growth in each individual microparticle with phase evolution. The crystallite size described in the previous section also increases as the sintering temperature increases, which is in good agreement with the tendency observed by FE-SEM (Figure 3). This indicates that the synthesized calcium aluminate phase (CAO) at the alumina microparticle surface is composed of crystalline material, forming polycrystalline particles since the calculated crystallite sizes are considerably smaller than the observed average grain sizes.

However, the presence of rare earth could play a relevant role in the nanostructure evolution. In this sense, the luminescence response will provide further pieces of evidence of the doping evolution and the micro-nano structure of each microparticle.

### 3.3. Photoluminescence Characterization and After Glow Properties

**Figure 4:**
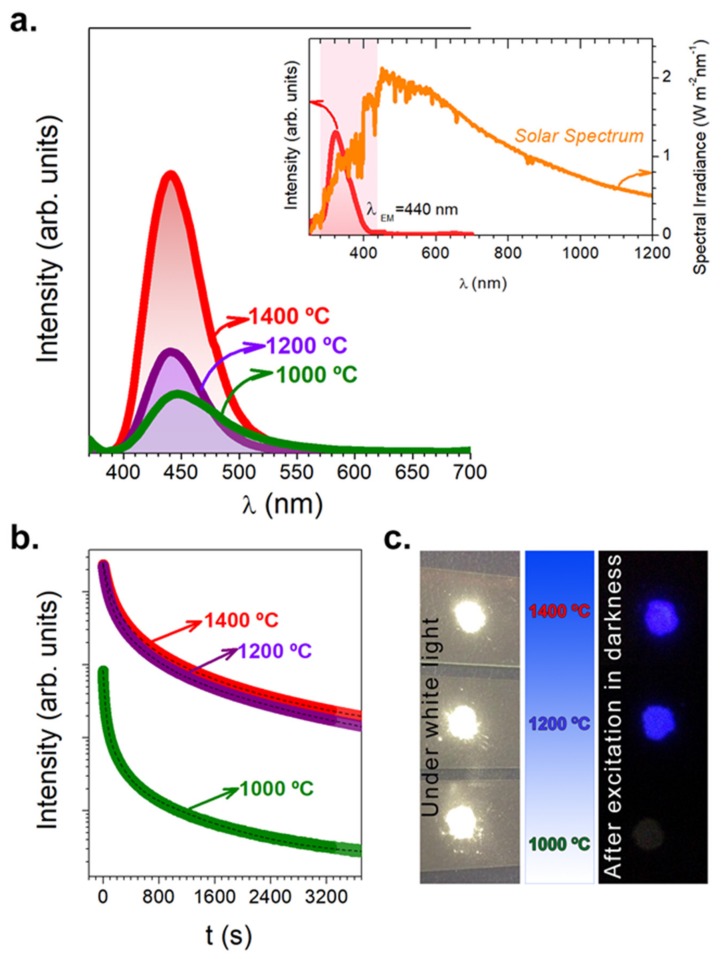
(**a**) Photoluminescence emission spectrum (λ_EXC_ = 365 nm) of synthesized CAO: Eu, Nd phosphors heated at 1000 (green line), 1200 (purple line), and 1400 °C (red-line) for 2 h in a 90N_2_-10H_2_ atmosphere, employing a salt/CAO molar ratio of 3:1. The inset shows the excitation spectrum fixing the emission at 440 nm (red-line) and the standard solar spectrum (American Society for Testing and Materials (ASTM) E-490) (orange line). (**b**) Afterglow decay curves of synthesized CAO: Eu, Nd phosphor heated at 1000 (green line), 1200 (purple line), and 1400 °C (red-line) for 2 h in 90N_2_-10H_2_, employing a salt/CAO molar ratio of 3:1 (**c**) Photographs of the CAO particles under white light excitation (left-image) and in dark after being activated for 10 min by a solar simulator (right-image).

Figure 4a shows the PL emission spectrum of the particles synthesized in the N_2_-H_2_ atmosphere. Eu dopant was incorporated as Eu_2_O_3_, in its trivalent oxidation state. Different techniques are described in the literature to reduce Eu^3+^ to Eu^2+^, and their stabilization is a non-trivial task. One possibility is to exploit the most commonly used H_2_ as a reduction agent. In our case, the annealing process carried out under a reductive atmosphere of 90N_2_-10H_2_ promotes the reduction of Eu^3+^ to Eu^2+^. Based on PL emission spectroscopy, it is straightforward to assess the presence of Eu^2+^ and Eu^3+^ emission centers. The Eu^3+^ emission is characterized by the narrow lines between 550 and 750 nm attributed to 4f→4f (^5^D_0_/^7^F_j = 0,1,2,3,4_) transitions. As shown in Figure 4a, the particles synthesized exhibit a broad emission band centered at 440 nm attributed to typical 4f^6^5d^1^→4f^7^ transitions of Eu^2+^ under the excitation at 365 nm. A shift in the luminescence band position related to the Ca_12_Al_14_O_33_ phase synthesized at 1000 °C can be explained by a small change in the crystal field effect on the Eu^2+^ ions because of the splitting. It is important to remark that the emission peak attributed to the Eu^2+^ cations [17] transition becomes more intense with an increase in the temperature (from 1000 to 1400 °C). The emission intensities are lower for the powder synthesized at 1000 °C, where the cubic phase Ca_12_Al_14_O_33_ is the predominant phase. At 1200 and 1400 °C, the emission intensity further increases. At 1400 °C, the single monoclinic phase of CaAl_2_O_4_ is steadily developed. As a first approach, the increment of the emission luminescence can be related to the phase composition. The inset in Figure 4a shows the excitation spectrum monitored at the 440 nm wavelength. This spectrum covers a broad spectral region from 273 to 418 nm, assuming that the phosphor can be activated in this range. The standard solar spectrum (ASTM E-490) is presented to check that effectively a range of energies (UV-B and UV-A) of the solar irradiation spectrum can be used to stimulate the particles. It is known that the maximum fraction effectively used by Si solar cells [18] is lower than the maximum fraction available in this range. Therefore, the employment of CAO: Eu, Nd material on the top of the solar panel can serve as persistent storage. [19,20] A comparison between the intensity obtained in CAO:Eu, Nd material and the commercial powder based on SrAl_2_O_4_:Eu, Dy (from Jinan Chenghao Technology Co., Ltd., Mingshihaoting, NO.12406 Jingshi East Road, Jinan, 250014, China) as received is shown in the Supporting Information (Figure S1).

Besides the intense and broad photoluminescent emission, the CAO: Eu, Nd particles demonstrate a long-lasting luminescence after the stoppage of UV irradiation. The density of trapped carriers plays a key role in the enhancement of the afterglow duration. Specifically, co-doping with Nd^3+^ results in a higher density of trapped carriers as compared with other rare earths [4,21].

Therefore, to prolong the afterglow, there must be empty energy levels available at shallow as well as deep locations in the bandgap. By the molten salt synthesis proposed here, the host can hold the luminescent center introduced, creating the required and distributed empty defects to prolong the blue luminescence. 

Measurements of the decay curve are one of the most useful ways to determine the duration of the luminescence. Figure 4b shows the persistent luminescence with decay time lasting from 0 s to 3600 s (1 h). The CAO: Eu, Nd particles were excited for 10 min, after that the excitation was cut off, and the afterglow curve was acquired. The profiles of the decay curves of the samples exhibit similar behavior. A multi-exponential decay profile was used to fit the experimental long afterglow decay data. Specifically, the observed afterglow decay curve was fitted by a quatri-exponential decay, following this equation:

(2) $$I=I_{1}+A_{1}\exp\frac{-t}{\tau_{1}}+A_{2}\exp\frac{-t}{\tau_{2}}+A_{3}\exp\frac{-t}{\tau_{3}}+A_{4}\exp\frac{-t}{\tau_{4}}$$

where *I*_1_ is the final intensity; *A*_1_, *A*_2,_ *A*_3,_ and *A*_4_ are constants; *t* is the decay time; and *τ*_1_, *τ*_2_, *τ*_3,_ and *τ_4_* are fitting parameters related to the decay rate of the phosphors. The fitting parameters are given in Table 2. 

The initial intensity of the sample thermally treated at 1000 °C is lower, and the intensity drops two orders of magnitude compared with the samples annealed at 1200 °C and 1400 °C for 1 h. This behavior can be related to the phase composition obtained in each thermal treatment. The presence of the monoclinic polymorph phase of CaAl_2_O_4_ at 1200 °C and 1400 °C has more ability to store and release the energy in comparison with Ca_12_Al_14_O_33_. These four temporal processes may be related to different types of trap centers or different levels of the same trap species. As the persistent mechanism of CAO: Eu, Nd is not yet established, to evaluate the decay rate of the fast (*τ_1_*), rapid (*τ_2_*), medium (*τ_3_*) and slow (*τ_4_*) exponential decay components, the steady-state contributions are quantified with *A_i_* × *τ_i_* products (*i* = 1 to 4). *A_i_* represents the amplitude of the exponential decay and *τ_i,_* the lifetime [22]. These values are acquired from the numerical fits, and by these calculations, the fractional contribution of each component can be obtained, according to the following expression:

(3) $$I\left(t\right)=\underset{i}{\sum }A_{i}e^{-t/\tau_{i}}$$

The fraction of each component in the multi-exponential decay is:

(4) $$S_{i}=\frac{A_{i}\cdot \tau_{i}}{\sum_{i}A_{i}\cdot \tau_{i}}\times 100$$

The contributions of medium (*τ_3_*) and slow (*τ_4_*) components have a higher weight in the decay process, which means that the persistence process is prolonged. 

An important remark is that CAO: Eu, Nd white powders are obtained under indoor illumination following the molten salt procedure in comparison with the dark-gray color characteristic of these long-lasting materials. The origin of the dark-gray color, instead of white, in our material and in other oxides synthesized under the N_2_/H_2_ atmosphere, is attributed to the presence of oxygen vacancies in the structure [23]. (Left image in Figure 4c). The right image (Figure 4c) exhibits the response of the CAO: Eu, Nd powders in darkness after cutting of the excitation.

### 3.4. Tuning the Functional Properties by Heat Treatment and Eu2+ Content

It is important to evaluate the intensity of the emission and a way to modulate it. As was previously shown, not only the crystallinity and the particle size are the parameters influencing the brightness. The fraction of the Eu^2+^ plays a key role in achieving higher intensities [23]. However, it is far from obvious to determine the fraction of both species from PL spectroscopy [23]. To do that, the XANES technique was employed, which can be considered as a powerful tool to investigate the valence states of the rare-earth dopants, i.e., europium. The significant difference in the absorption energy of the two oxidation states can serve as a signature (6971.8 eV for Eu^2+^ and 6979.5 eV for Eu^3+^). 

**Figure 5:**
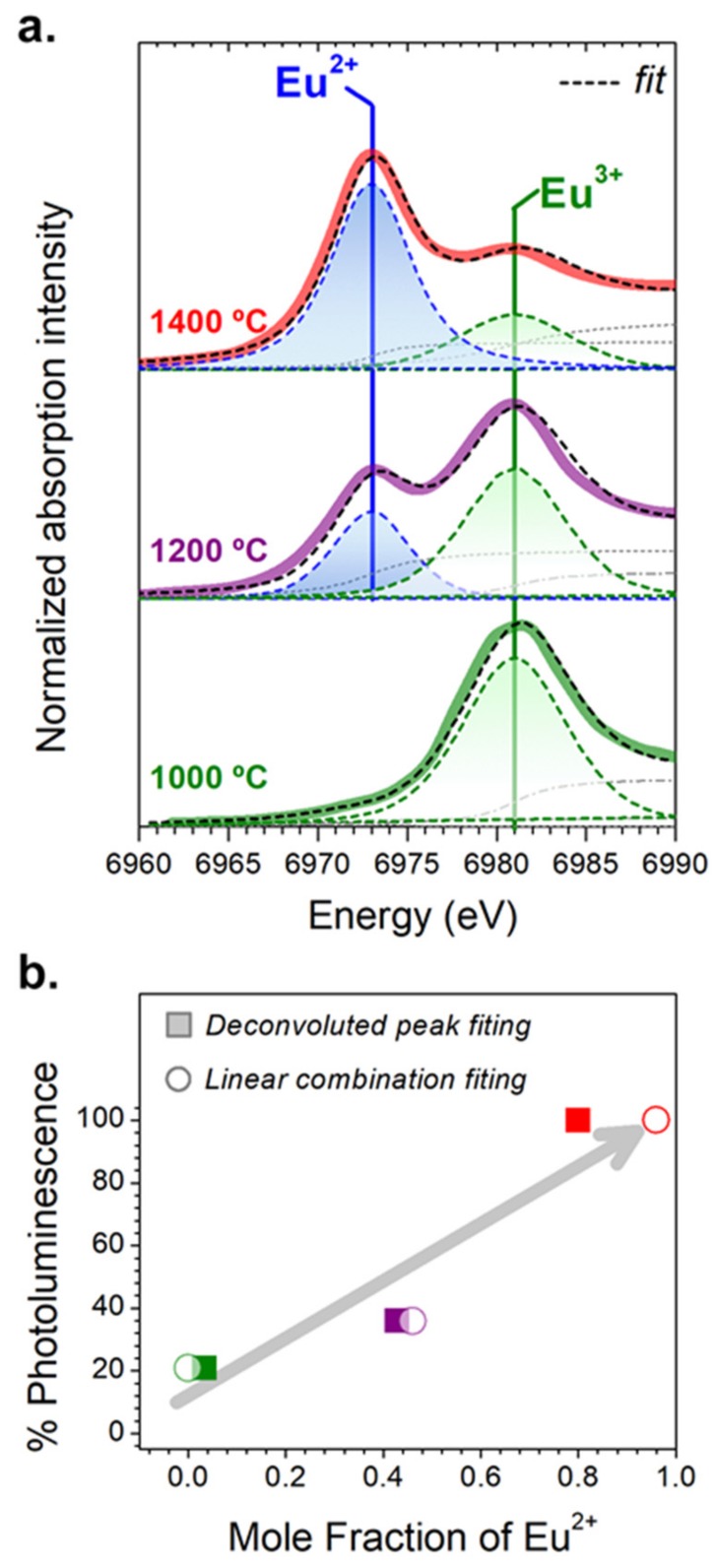
(**a**) X-ray absorption near-edge structure spectroscopy (XANES) measurements at the Eu L_3_-edge of the particles synthesized at 1000 (green spectrum), 1200 (purple spectrum), 1400 °C (red spectrum). The fitting (black dashed curve) and the individual components (green and blue dashed curves) of the deconvoluted absorption peaks using pseudo-Voigt and arctangent step (gray dashed curves) functions are shown in the same graph. (**b**) % of photoluminescence (PL) intensity of CAO particles synthesized at 1000 (green square and circle), 1200 (purple square and circle), 1400 °C (red square and circle) as a function of the Eu^2+^ fraction calculated by the deconvoluted peaks and by a lineal combination fitting. The % PL intensity is referred to as the value obtained for the sample synthesized at 1400 °C.

As Figure 5a depicts, an increase in the thermal temperature results in the domination of the absorption peak of the divalent Eu cations. It is worth it to remark that a considerable fraction of Eu ^3+^ can be detected in the particles of CAO, but the concentration is not reflected by the PL spectrum. 

To determine the concentration ratio of Eu^2+^ and Eu^3+^ in the CAO: Eu, Nd particles, two methods were assessed. Several studies quantify the relative abundance of Eu^2+^ and Eu^3+^ ions in the samples by means of a linear combination fitting of standards at the Eu L_3_-edge absorption. In this case, an absorption signal combination of Eu_2_O_3_ and EuI_2_ standards is performed using the Athena software. The fitting values for Eu L_3_-edge XANES spectra analyzed by a linear combination of Eu_2_O_3_ and EuI_2_ references are compiled in Table 3. However, these analyses do not consider the transition probabilities of the two states (2^+^ and 3^+^). 

Previous studies suggest that the peak amplitude related to Eu^2+^ L_3_-edge resonance is 1.5 times smaller than that corresponding to the Eu^3+^ resonance peak [24,25]. Taking this into account, the deconvoluted peak fitting of the white line (WL) at the Eu L_3-_edge was done by using a pseudo-Voigt and an arctangent step function for each peak of the absorption spectra [25,26] and employing the mole ratio M_Eu_ through M_Eu_ = RA_Eu_ [25,27]. The R-value is 1.5, and the A_Eu_ represents the ratio between the Eu^2+^ and Eu^3+^ WLs. Figure 5b shows the best fitting done by modeling the experimental data, and the results taking the reported transition probability difference are summarized in Table 4. It should be noted that during the XAS experiments, no modifications in the average valence of the samples were identified by the X-ray irradiation.

Despite the fact that from the linear combination fitting, the resulting Eu^2+^ percentage for the sample thermally treated at 1000 °C shows that Eu^2+^ is not present, the results obtained by the deconvoluted peak fitting and by PL spectroscopy reflect a low content of Eu^2+^. It means that in spite of XANES analysis exhibiting an insignificant fraction of Eu^2+^ cations, this quantity is enough to allow the blue emission of powder. In any case, further research is required to optimize the reduction process via the molten salt route, in order to increase the emission intensity. For samples prepared at 1200 °C and 1400 °C, the Eu^2+^: Eu^3+^ fraction is calculated to be around 0.5:0.5 and 1:0, respectively, by both approaches, which agrees with the PL response. 

Figure 5b exhibits the percentage of PL against the fraction of Eu^2+^ calculated from XANES spectra fitting by the deconvoluted peak and linear combination method. The relative amounts of Eu^2+^ determined by two fitting approaches show that the % of PL, calculated for each sample, taking as 100% the PL intensity obtained for the sample sintered (frittage) at 1400 °C, increases when the Eu^2+^ fraction increases, following a linear trend. Even so, the tendency is similar in both XANES fitting. The XANES results given from the fitting at the whiteline (WL), considering the transition probabilities of the two Eu^2+^ and Eu^3+^ states by the deconvoluted peak fitting or by the linear fitting of absorption signal with the Eu_2_O_3_ and EuI_2_ standards, provide the relative abundance of Eu^2+^ and Eu^3+^. This quantification cannot be assessed by taking into account only the results obtained by PL spectroscopy, showing that the joint characterization by both XANES and PL is necessary to evaluate and optimize the reduction of Eu species.

## 4. Conclusions

The reported method based on assisted molten salt flux evidences the production potential of the afterglow calcium alumina blue emitters. Analysis of the reaction mechanism confirms that the dissolution-diffusion transport process (template mechanism) prevails over the dissolution-precipitation mechanism. This novel approach proposed for the synthesis of the Ca_12_Al_14_O_33_ and CaAl_2_O_4_ phases doped with Eu and Nd, controls the shape, morphology, and homogeneity of the platelets obtained. Overall, the micro-particles are individual ceramic particles composed of fully-sintered grains. The nanostructure of each micro-particle evolves with the synthesis temperature, favoring both the CaAl_2_O_4_ phase formation and the dopant incorporation into the structure. Remark that micro-particles are white in appearance, a fact that solves the problem of the dark gray coloring on aluminates synthesized in reducing atmospheres. Our observations demonstrate that persistent phosphors based upon calcium-aluminate compounds can be effectively activated by UV light and sunlight irradiation. The materials can work independently after being charged by artificial or solar light that is quite useful for practical applications. As regards the physical origin of the optical response, the results indicate that the control of the Eu^2+^/Eu^3+^ ratio (that is, find an optimal ratio of the chemical substituent) by suitable synthesis conditions is the key factor for enhancing the luminescence response. It is increasingly recognized that although calcium-aluminate systems have been studied extensively, the optimization of the Eu reduction by annealing conditions can open new paths in order to obtain the highest emission intensities.

## Figures and Tables

**Table 1:** Average particle size, d_50_, and d_10_ and d_90_ values of synthesized CAO: Eu, Nd phosphor heated at 1000, 1200 and 1400 °C for 2 h in 90N_2_-10H_2_, employing a salt/CAO molar ratio of 3:1.

	Particle Size		
Sample	d_10_(μm)	d_50_(μm)	d_90_(μm)
1000 °C	2.6	7.8	18.1
1200 °C	2.9	8.8	19.0
1400 °C	5.9	17.0	43.7

**Table 2:** The fitting results of the decay parameters and the fractional contributions of the lifetime components (i.e., *τ_1_*, *τ_2_*, *τ_3_*, and *τ_4_*). *A_1_*, *A_2_*, *A_3_*, and *A_4_* are constants, and *S_1_*, *S_2_*, *S_3_*, and *S_4_* represent the % of each component.

Sample	τ_1_ (s)	τ_2_ (s)	τ_3_ (s)	τ_4_ (s)	A_1_	A_2_	A_3_	A_4_	% S_1_	% S_2_	% S_3_	% S_4_
1000 °C	7.7	31.6	147.0	871.3	347281	328287	117781	27059	4.9	19.2	32,1	43.7
1200 °C	31.0	101.3	339.8	1386.5	9320400	8291800	3862010	1191890	7.1	20.5	32.0	40.4
1400 °C	61.2	216.3	735.2	3064.6	14081900	7358350	2473830	559904	14.4	26.6	30.4	28.7

**Table 3:** Results of the linear combination fitting at the Eu^2+^ and Eu^3+^ L_3_-edge WLs for the XANES spectra of the CAO: Eu, Nd particles synthesized at 1000, 1200, 1400 °C for 2 h in a 90N_2_-10H_2_ atmosphere, employing a salt/CAO molar ratio of 3:1, alumina platelets with an average particle size d_50~_6 μm, and using an Al_2_O_3_/CAO molar ratio of 1.

Sample	Species	Fraction
1000 °C	Eu^2+^	0.00 (2)
	Eu^3+^	1.00 (2)
1200 °C	Eu^2+^	0.54 (3)
	Eu^3+^	0.46 (3)
1400 °C	Eu^2+^	0.96 (4)
	Eu^3+^	0.04 (1)

**Table 4:** Fitting results of Eu^2+^ and Eu ^3+^ L_3_-edge WLs from CAO particles synthesized at 1000, 1200, 1400 °C for 2 h in a 90N_2_-10H_2_ atmosphere, employing a salt/CAO molar ratio of 3:1, alumina platelets with an average particle size, d_50~_6 μm, and using the Al_2_O_3_/CAO molar ratio of 1 by deconvoluted peak fitting using pseudo-Voigt and arctangent step functions.

		Edge Resonance				Edge Step			
Sample	Species	Height Arb.u.	Position eV	FWHM	Area	Height Arb.u.	Position eV	Fraction	R-Factor
1000 °C	Eu^2+^	0.45	6971.0	12.00	0.45	0.113	6971.0	0.03(1)	0.00232
	Eu^3+^	19.70	6981.0	13.97	19.70	0.649	6981.0	0.97(1)	
1200 °C	Eu^2+^	6.128	6971.0	13.34	6.128	0.559	6971.8	0.43 (1)	0.00119
	Eu^3+^	12.29	6981.0	9.51	12.29	0.311	6979.5	0.57 (1)	
1400 °C	Eu^2+^	15.20	6971.0	9.88	15.20	0.319	6971.8	0.83 (6)	0.00150
	Eu^3+^	5.69	6981.0	14.54	5.69	0.572	6979.5	0.17 (6)	

## Supplementary Materials

The following are available online at https://www.mdpi.com/2079-4991/9/10/1473/s1.
