# Supplementary material for: Towards Blue Long-Lasting Luminescence of Eu/Nd-Doped Calcium-Aluminate Nanostructured Platelets via the Molten Salt Route
Source: Nanomaterials (Basel). 2019 Oct 16;9(10):1473. doi: 10.3390/nano9101473 (PMC6836158; doi:10.3390/nano9101473)
Supplement: Supplementary file 1 [file nanomaterials-09-01473-s001.pdf]

Supplementary Material

Towards blue long-lasting luminescence of Eu/Nd doped calcium-aluminate nanostructured platelets via molten salt route

Rocío Estefanía Rojas-Hernandez[*, †, ‖], Fernando Rubio-Marcos[†], Aida Serrano[†], Eduardo Salas[*], I. Hussainova[‖], José Francisco Fernandez[†]

[†] *Electroceramic Department, Instituto de Cerámica y Vidrio, CSIC, Kelsen 5, 28049, Madrid, Spain*

[‖] *Department of Materials Engineering, Tallinn University of Technology, Ehitajate 5, 19180 Tallinn, Estonia*

[*] *Spanish CRG BM25 SpLine beamline, The European Synchrotron, 38043 Grenoble, France, and Instituto de Ciencia de Materiales de Madrid, CSIC, 28049, Madrid, Spain.*

Supplementary Information 1:

Figure S1 shows the PL emission spectrum of the particles synthesized in N_2-H_2 atmosphere heated at 1000 (green-line with diamonds), 1200 (purple-line with circles), and 1400 ºC (red-line with squares) compared with the commercial powder as received based on $SrAl_2O_4$:Eu, Dy material (SAO-Reference).

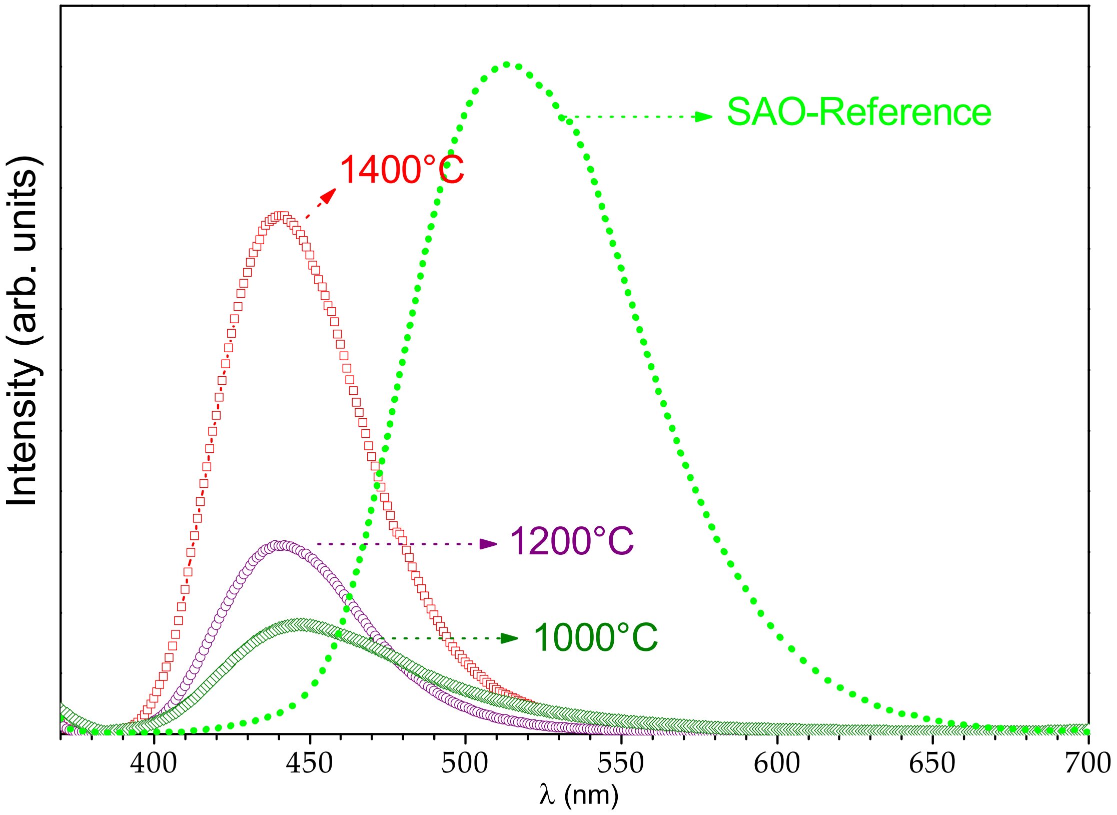

Figure S1 Photoluminescence spectra of the synthesized CAO: Eu, Nd phosphor heated at 1000 (green-line with diamonds), 1200 (purple-line with circles), and 1400 ºC (red-line with squares) for 2 hours in $90N_2$-$10H_2$, employing a salt/CAO molar ratio of 3:1 in comparison to a commercial powder (from Jinan Chenghao Technology Co., Ltd) as received.
